# Production and Characterization of *H. perforatum* Oil-Loaded, Semi-Resorbable, Tri-Layered Hernia Mesh

**DOI:** 10.3390/polym17020240

**Published:** 2025-01-19

**Authors:** Özlem Eğri, Feyza Güneş, Sinan Eğri

**Affiliations:** 1 Department of Mechanical Engineering, Faculty of Engineering and Architecture, Tokat Gaziosmanpaşa University, 60250 Tokat, Türkiye; ozlem.egri@gop.edu.tr; 2 Institute of Graduate Studies, Bioengineering Division, Tokat Gaziosmanpaşa University, 60250 Tokat, Türkiye; gunessfeyzaa@gmail.com; 3 Department of Chemistry, Faculty of Science and Letters, Tokat Gaziosmanpaşa University, 60250 Tokat, Türkiye

**Keywords:** hernia mesh, *H. perforatum* oil, electrospinning, chlorinated polypropylene, polycaprolactone, polyethylene glycol

## Abstract

Hernia repair is the most common surgical operation applied worldwide. Mesh prostheses are used to support weakened or damaged tissue to decrease the risk of hernia recurrence. However, the patches currently used in clinic applications have significant short-term and long-term risks. This study aimed to design, produce, and characterize a three-layered semi-resorbable composite hernia mesh using the electrospinning technique, where the upper layer (parietal side) was made of non-resorbable polypropylene (PP-Cl) fibers, the partially resorbable middle layer was made of PP-Cl and polycaprolactone (PCL) fibers, and the fully resorbable lower layer (visceral side) was made of *H. perforatum* oil-loaded polyethylene glycol (PEG) fibers. The extracellular matrix-like fibrous structure of the patches provided low density and high porosity, minimizing the risk of long-term foreign body reactions, and the hydrophilic/hydrophobic character of the surfaces and the detected swelling rates supported biocompatibility. The patches exhibited mechanical properties comparable to commercially available products. Controlled release of therapeutic oil could be achieved from the oil-integrated patches due to the dissolution of PEG in the acute process. In vitro cell culture studies with the L929 mouse fibroblast cell line revealed that the meshes do not have a cytotoxic nor a biomaterial-induced necrotic effect that will induce apoptosis of the cells. The visceral side of the meshes exhibited non-adherence of cell-like structures to the surface due to the dissolution of PEG. The composite hernia patches were concluded to reduce the risk of adhering to internal organs in the hernia area, have the potential to be used in in vivo biomedical applications, and will support the search for an ideal hernia mesh that can be used in the treatment of abdominal hernias.

## 1. Introduction

Hernia operations are among the most common surgical operations performed in the world due to the ever-increasing number of abdominal surgical operations and the increase in factors that cause the risk of hernia, such as obesity [1]. Hernia is the displacement of a tissue or an organ, such as the intestine, from the biological walls surrounding the area, causing swelling under the skin [2]. Hernias usually occur in the abdominal and groin areas [3]. The therapy varies depending on the type and size of the hernia. It is challenging to prevent recurrence, especially in large hernias. In this case, the hernia area must be supported with a prosthesis [4]. Polypropylene (PP) patches, first used in the 1960s, have been the most widely used synthetic biomaterials in hernia therapy [3]. One of the most essential advantages of using PP patches in hernia repair is the quick integration of the host tissue into the prosthesis [5]. However, one of the most important disadvantages of PP patches is that they tend to adhere to internal organs and tissues [3]. PP is not an absorbable, biodegradable material. Therefore, due to shrinkage of the biomaterial in the patch region in the long term, issues such as loss of compliance with the abdominal wall or inflammation in the patch region may occur. To solve these problems, low-density PP patch designs have been studied as an alternative to reduce the amount of foreign matter [5,6]. However, although attempts have been made to preserve tissue integration and abdominal wall compatibility by reducing the risk of shrinkage post-implantation, the problems have not been resolved [7,8]. Alternatively, producing patches using the electrospinning technique gives the patch a high degree of porosity and reduces the patch density while preserving mechanical strength. However, the spinning of PP by the electrospinning technique has been limited due to its high solvent and electrical resistivity [9,10,11]. The addition of polar halogen atoms to the apolar backbone of PP has been reported to increase the polarity and compatibility of the polymer even at low concentrations [12].

Polycaprolactone (PCL) is a semicrystalline, biodegradable, and biocompatible polymer studied in various biomedical applications, even in the prevention of abdominal adhesions, and it has been reported that PCL electrospun membranes reduced adhesions in the abdominal cavity in rat model [13].

Hypericum perforatum (St. John’s Wort) oil has wound-healing properties thanks to its active ingredients, such as hypericin and hyperforin [14]. There are also several studies in the wound healing literature in which *H. perforatum* oil is used directly or integrated into wound dressing materials [15,16].

The important features sought for an ideal patch are not interacting with the surrounding tissue in in vivo applications (inertness), being resistant to infection or even preventing it (antimicrobial properties), maintaining sufficient tensile strength in the long term to prevent hernia recurrence, fast adaptation to the host tissue (biocompatibility), showing enough mechanical strength to adapt to the tension due to body movements in the surrounding tissue such as abdominal muscles, etc. (flexibility), not being tumorigenic (anti-carcinogenic), supporting healing and functionality during the process of regaining the natural respiratory movements of the abdominal wall, and keeping the patch density at an optimal level that will not cause foreign body reaction in the short and long term [4].

In this study, biocompatible, elastic, three-layered, semi-absorbable patches that will support the accelerated healing of hernia wounds with an original design were produced, and patch performance was evaluated using various in vitro characterization methods. The primary reason for designing the patches as semi-absorbable is to minimize the risk of hernia recurrence and foreign body reaction in the long term. To our knowledge, this study integrated a therapeutic herbal oil into hernia patches for the first time.

## 2. Materials and Methods

The *H. perforatum* oil produced by the cold press method and with 100% purity used in this study was supplied by Awe Cemre Laboratories (Tokat, Turkey). PEG, with a number average molecular weight of 10 kDa (Merck, Darmstadt, Germany), PCL, with a number average molecular weight of 80 kDa (Sigma-Aldrich, St. Louis, MO, USA), and PP-Cl, with a number average molecular weight of 65 kDa and 29–32% chlorine mass fraction (Mark Zhang Shanghai Sunking Industry Incorporation, Shanghai, China), polymers were also purchased. Chloroform (Merck, Darmstadt, Germany), methanol (Merck, Darmstadt, Germany), and tetrahydrofuran (Sigma-Aldrich, Steinheim, Germany) solvents were also purchased and used as received for the preparation of polymer solutions.

### 2.1. Production of Three-Layered Composite Hernia Mesh

**Figure 1:**
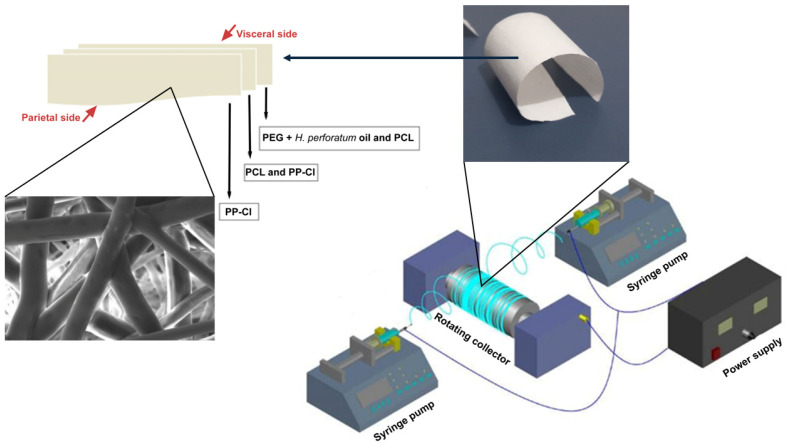
Schematic representation of tri-layered hernia mesh production.

The production of three-layered semi-absorbable composite hernia patches was achieved by the counter-direction electrospinning setup (Figure 1). The lower layer (visceral side), where the hernia operation was performed and would be in contact with the wound area, was formed by counter-electrospinning the PEG solution containing *H. perforatum* oil (herbal oil) and the PCL solution and collecting them on the aluminum foil surface covered on the rotating drum collector. Then, while the PCL fibers were collected on the collector surface during the electrospinning of the PCL solution, the PP-Cl solution started to be electrospun instead of the PEG solution containing oil. The thin middle layer formed by counter-spinning the PCL and PP-Cl solutions for a certain period was completed, and then the PP-Cl solution was electrospun alone. Thus, the upper layer (parietal side), consisting of only PP-Cl fibers, was formed after a certain period, and patch production was finalized. All polymer solutions used in the construction of all layers of the patch were evaluated and optimized with different solutions at different concentrations and under different dissolution parameters. Many parameters, such as flow rates from syringe pumps, total flow periods, voltage values to be applied, distances between syringes and collector, ambient temperature and humidity, etc., are known to affect the thickness of fiber structures and the surface properties of the fibers and were optimized individually [12,17,18]. In this context, two different patches were prepared with PCL (15% *w*/*v*), PEG (4% *w*/*v*), and PP-Cl (20% *w*/*v*) solutions, namely, ‘patch 0’ without *H. perforatum* oil and ‘patch 1’ containing *H. perforatum* oil. The PEG polymer solution was prepared by adding herbal oil to the chloroform solvent (10% *v*/*v*). The distance between the rotating drum collector and the tip of the syringe, to which the PEG polymer solution was added, was determined as 7 cm. The solution concentration of the chloroform/methanol mixture (5:1) used to dissolve the PCL polymer was determined as 15% (*w*/*v*), and the distance between the rotating drum collector to the tip of the syringe containing the PCL polymer solution was determined as 13 cm. The PP-Cl polymer was dissolved in a tetrahydrofuran (THF) solvent on a magnetic stirrer for 4 h. The solution concentration of the THF used to dissolve the PP-Cl polymer was determined as 20% (*w*/*v*), and the distance between the syringe tip and the rotating drum collector was determined as 17 cm.

Firstly, for the first layer of hernia patches, the PCL polymer solution was electrospun at a flow rate of 1.5 mL/h and the PEG polymer solution at a rate of 3 mL/h under a potential difference of 15 kV. After 1 h, the flow rate of the PP-Cl polymer solution was adjusted to 2 mL/h. After the PEG polymer solution was depleted, the PP-Cl polymer solution was added instead, and the middle layer was spun at a flow rate of 2 mL/h under a potential difference of 16 kV with the PCL polymer solution for approximately 1.5 h. After the PCL polymer solution was depleted, the PP-Cl polymer solution was electrospun alone for 1 h, and the production of the third and last layer was completed. After the electrospinning process, the patches were labeled and left to dry at room temperature for at least two days to remove the residual solvent and stabilize the patches. The resulting patches are shown in Figure S1. The produced patches have the appearance of white textured paper with a width of approximately 10 cm and a length of roughly 29 cm.

### 2.2. Hernia Mesh Characterization

#### 2.2.1. Chemical Structure Characterization

To check whether there is any change in the chemical structure of the biocompatible polymers forming the patch due to the interaction with the solvent and electrospinning conditions, such as high potential difference, etc., the FTIR-ATR (Fourier Transform Infrared Spectroscopy–Attenuated Total Reflection, Jasco, FT-IR/4700, Tokyo, Japan) spectra of the polymers (PP-Cl, PCL, PEG), *H. perforatum* oil, and the oil-free patch (patch 0) and oil-loaded patch (patch 1) were taken separately and comparatively examined. The patches and polymers were carefully placed on a diamond disk with a few millimeters diameter and fixed to the disk surface under pressure. Measurements were taken by recording the absorption of the infrared (IR) beam sent to the surface by the material and the IR intensity reflected from the crystal.

#### 2.2.2. Morphological Analysis by Scanning Electron Microscope (SEM)

Circular sections of 7.5 mm diameter were taken from the patches (patch 0 and patch 1; top and bottom surfaces), and the patch surfaces were coated with gold (Au) nanoparticles under vacuum for 10 min to make the patch sections conductive. Then, SEM (ZeissEvo Ma10, Carl Zeiss AG, Oberkochen, Germany) images of the patches were taken at different magnifications, and their surface morphologies were investigated.

#### 2.2.3. Density and Porosity

Five square sections, prepared with 1 × 1 cm dimensions, were taken from the patches, and the density (*ρ*) and average specific volume (cm^3^/g) of the patches were calculated using the weight (g) and volume (cm^3^) values. Then, the % porosity values were calculated with the help of the formula (Equation (1)) in accordance with the literature [19].

Porosity (%) = [(V_m_ − V_0_)/V_m_] × 100, (1)

where V_0_ represents the specific volume (cm^3^/g) for the pure polymeric film and V_m_ represents the specific volume (cm^3^/g) values for the electrospun polymeric patches

#### 2.2.4. Water Contact Angle

The contact angle test measurements of the patches were performed using a water contact angle device (Attension, Biolin Scientific AB, Gothenburg, Sweden) following the ASTM-D7334-2013 standard. First, 4 μL of deionized water was dropped onto the bottom and top layers of patch 0 and patch 1, respectively, and a total of 12 samples were studied with triplicates. An image of the water drop on the patch surface was captured using a camera. The images were examined, and the contact angle value was determined for each section. The average contact angle was calculated by taking the average angle values of the patch surface with the water droplet. The hydrophilic/hydrophobic characters of the bottom and top surfaces of the oil-loaded (patch 1) and oil-free (patch 0) patches were evaluated.

#### 2.2.5. Moisture Retention Capacity (Swelling Tests)

The water uptake capacities (wettability) of the patches were calculated using the formula given in Equation (2) using the dry weights of circular patch sections with a diameter of 7.5 mm and their wet weights after being kept in phosphate buffer solution (37 °C, pH 7.4) for specific periods (3, 5, 6, 9, 10, 15, 20, 25, 30, 45, 60, 75, 90, 110, 130, 150, 180 min) [20].

Swelling ratio (%) = [(Wt − W0)/W0] × 100(2)

where W_0_ and W_t_ represent the dry and wet weights of the patches, respectively.

#### 2.2.6. Time-Dependent Mass Loss

To measure the mass loss of the patches over time, 5 sections of 2 cm × 2 cm were taken from patches 0 and 1 each day. After the initial weights of the sections were measured, they were placed in PBS (pH 7.4, 37 °C) solution and then put into an oven. Five samples were taken from each patch group daily and weighed again after drying in the oven. The experiment was continued for 40 days for both experimental groups. The percentage remaining mass was calculated using Equation (3) based on the pre-experiment dry weight (Wi) and post-experiment dry weight (Ws) values of the patch sections, and a graph was obtained.

Mass remaining (%) = (W_s_/W_i_) × 100 (3)

#### 2.2.7. Controlled Release

To investigate the release profile of the oil from the patch under in vitro conditions, circular sections of 7.5 mm diameter were taken from the patches (patch 0, patch 1), and the amount of *H. perforatum* oil released from the patches in PBS solution (37 °C and pH 7.4) with time was measured by taking the absorbance values using a spectrophotometer. It was stated that the wavelengths at which *H. perforatum* oil showed maximum absorbance were in the UV region at 270–330 nm [21] and in the visible region at 550–590 nm [14]. Considering these values, a series of studies were conducted to determine the wavelength. The highest peak value of 1 μL/mL *H. perforatum* oil solution was determined to be 225 nm, and measurements were taken with a spectrophotometer (BOECO S-30 Spectrophotometer, Hamburg, Germany). Controlled release studies were evaluated on empty patches (patch 0) and oil-loaded patches (patch 1). Thus, we aimed to understand whether the polymer (PP-Cl, PCL, PEG) added to the patch structure was released into the environment and caused an interference in the absorbance value. Repeated control samples were also examined on empty patches at 225 nm, and no change was observed in the measurements. It was assumed that all values obtained from these results were due to the release of *H. perforatum* oil. For 20 days, 5 different samples were prepared under the same conditions daily, and the absorbance values of the samples taken from the PBS solution containing the patch sections were taken every 24 h. The absorbance values of the PBS solutions containing *H. perforatum* oil prepared at known concentrations were used in the UV spectrophotometer to create a concentration-dependent absorbance graph (calibration curve). Thus, the absorbance values of the samples taken from the PBS solutions containing oil at unknown concentrations were converted to solution concentrations with the help of the calibration graph, and a time-dependent concentration graph was created. With the help of the obtained graph, the release profile of *H. perforatum* oil, depending on the dissolution of PEG fibers in the visceral layer, was determined.

#### 2.2.8. Mechanical Strength

A tensile testing device (Autograph AGS-X, Shimadzu, Kyoto, Japan) was used in accordance with EN ISO 13934-1:2013 to determine the mechanical strength of the produced three-layer composite patches. For this purpose, the samples to be tested were cut into 0.5 cm × 10 cm dimensions and placed between the device sections. The measurements were made at room temperature with different forces between 1.5 and 4.5 N and a constant tensile speed of 50 mm/min. During the test, the tensile stress (stress) versus strain values (strain) of the patch sections, and, as a result, the tensile strength, yield point, and elasticity modulus (Young’s modulus) values, were calculated together with the elongation at break. Five samples were tested for each patch group (patch 0, patch 1), and the average values were calculated.

#### 2.2.9. Cytotoxicity (% Cell Viability, % Apoptosis, % Necrosis)

Cytotoxicity tests, which are an important step for the evaluation of the biocompatibility of the produced hernia patches, were performed. For this purpose, the MTT (3-(4.5-dimethylthiazol-2-yl)-2.5-diphenyltetrazolium bromide) test, which is a widely accepted intracellular-specific staining test in the literature, was conducted in accordance with the ISO 10993-5 standard [22]. First, 96-well plates were seeded with 5 × 10^5^ L929 fibroblast cells in each well and then incubated in the appropriate medium (1% antibiotic (penicillin), 10% fetal bovine serum (FBS), and DMEM L-glucose (low glucose)) at 37 °C in a CO_2_ (5%) environment for 24 h. The studies were conducted for a total of 5 groups, including the group containing only *H. perforatum* oil, the *H. perforatum* oil-free (patch 0) and loaded patch (patch 1) evaluation groups, the negative control group consisting of cells not interacted with any material/substance, and the positive control group consisting of cells interacted with DMSO, a solvent with known toxic effects, with more than 4 samples in each group, and the results were evaluated comparatively. The tested samples were sterilized under UV light for 30 min before cell culture. After 1 day of incubation, 50 μL of tetrazolium salt (MTT) solution was added to each well, and the cells cultivated in the wells for 2 h interacted with the MTT salt. After incubation, each well’s optical density (OD) values were recorded at 570 nm with an ELISA plate reader, and the %viability values were calculated using Equation (4).

Cell viability (%) = (OD_measued_/OD_control_) × 100 (4)

To determine apoptotic and necrotic cells, L929 mouse fibroblast cells were cultivated in an appropriate medium (1% antibiotic (penicillin), 10% fetal bovine serum (FBS), and DMEM L-glucose) and proper cell culture conditions (37 °C, 5% CO_2_). A total of 10^4^ cells were seeded in each well together with sterile samples and then 200 μL of the sterile medium was added. After the medium was discarded from the cells incubated for 1 day, the cells adhering to the surface without the addition of medium were stained using the double staining technique. After adding 150 μL of DoubleStain to each well, the plate was wrapped in aluminum foil, and the cells were incubated with the stain in the incubator for 15 min. After incubation, apoptotic and necrotic cells were counted, and % apoptosis/necrosis values were calculated.

#### 2.2.10. Investigation of Cell Adhesion to the Visceral Layer Surface of Patches

One of the significant disadvantages of hernia mesh application is the risk of the mesh adhering to the internal organs, especially on the visceral side, where there is intensive regeneration due to tissue healing after surgery [3]. New-generation composite patches are generally types of patches that have different surfaces on the lateral and visceral sides, with the addition of a biodegradable layer accompanying the nondegradable layer, providing mechanical strength and less risk of adhesion to tissue on the visceral side [23]. Soluble PEG fibers were used to prevent cell adhesion to the visceral side of the patches, and therapeutic herbal oil (*H. perforatum*), known to support wound healing, was integrated into the PEG fibers of patch 1. Cell adhesion on the visceral side in contact with the hernia region was investigated by taking SEM images after L929 fibroblast cells were added to the surface in a suitable medium and incubated for 2 days. For this purpose, L929 fibroblast cells were incubated in a suitable medium (1% antibiotic (penicillin), 10% fetal bovine serum (FBS), and DMEM L-glucose) at 37 °C and 5% CO_2_ conditions. After incubation, the medium was discarded, and the confluent monolayer cells were removed from the surface with trypsin-EDTA (0.25% Trypsin, 0.02% EDTA). The suspended cells were counted and placed in 24-well plates with the medium, each well containing 1 × 10^5^ cells (30 μL). Circular sections with a diameter of 0.75 cm taken from the patches were sterilized by exposure to UV light for 15 min and then placed in separate wells so that the visceral side would contact the medium containing the cells. After the inoculation, 200 μL of the medium was added to the wells and incubated at 37 °C and 5% CO_2_ for 2 days. After incubation, the patches were placed on a separate plate and left to dry. Then, the sections were placed on the SEM platform with the visceral side up and coated with palladium for 15 min in the Polaron SC500 Sputter Coater device (Quorum Technologies Ltd., Laughton, East Sussex, UK). Finally, scanning electron microscope (JEOL JSM-5600 SEM, Tokyo, Japan) images of the patch surfaces were taken to determine whether there was a cell-like structure adhering and/or proliferating on the visceral surfaces.

## 3. Results

### 3.1. Chemical Structure Characterization

**Figure 2:**
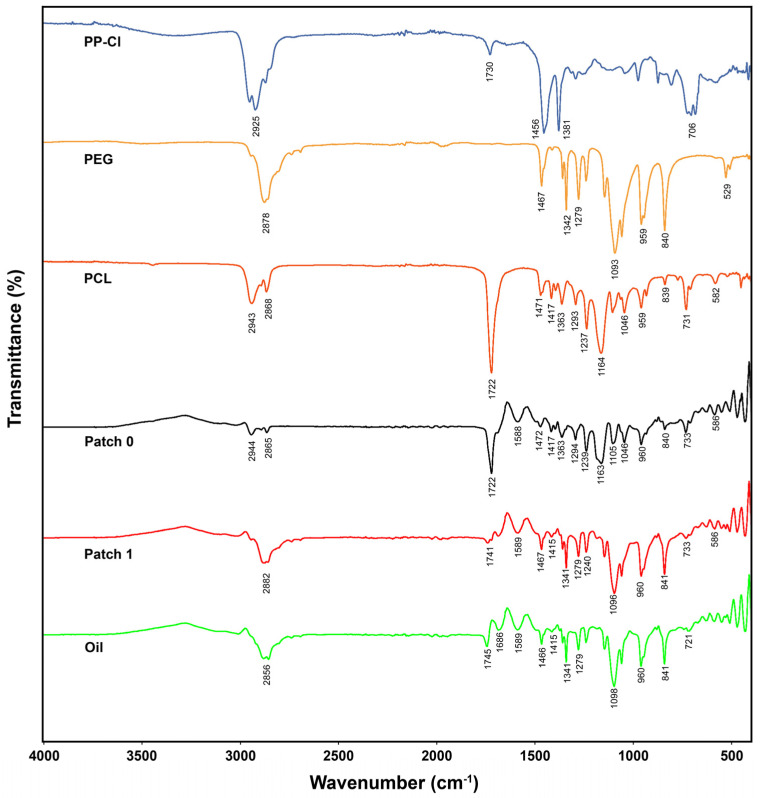
ATR-FTIR spectra of patches (0 and 1), polymers used in the production of patches (PP−Cl, PCL, PEG), and *H. perforatum* oil.

FTIR spectra of the polymers used in the hernia patch (PEG, PCL, PP-Cl), *H. perforatum* oil, and patches (patch 0 and patch 1) were taken separately. The interactions of the polymers with the solvent and whether working under high voltage during electrospinning resulted in any changes in the chemical structures of the polymers were investigated by comparative evaluation of the spectra (Figure 2). When the FTIR spectrum was examined, the C-H stretching peaks of PP-Cl, PEG, PCL, and *H. perforatum* oil were observed at wave numbers of 2925 cm^−1^, 2878 cm^−1^, 2943 cm^−1^–2868 cm^−1^, and 2856 cm^−1^, respectively [15,24]. Similarly, it was observed that the C-H stretching peaks for patch 0 and patch 1 were at 2865 cm^−1^–2944 cm^−1^ and 2882 cm^−1^, respectively. The peaks observed in the range between 1472 cm^−1^ and 1341 cm^−1^ in all these spectra are the peaks belonging to C-H bending.

The specific C-Cl peak of PP-Cl was observed at 703 cm^−1^ in the spectrum for PP-Cl only. This peak is slightly to the left at the 733 cm^−1^ wavenumber in the patch 0 and patch 1 spectra [24]. This is because the C-H bending peaks in the PCL (731 cm^−1^) and aromatic ring of the oil (721 cm^−1^) are at close wave numbers. Peaks belonging to the carbonyl (C=O) group were observed at 1722 cm^−1^ for PCL and the 1745 cm^−1^ wavenumber for *H. perforatum* oil, and it was seen slightly further to the left at a wave number of 1741 cm^−1^ for patch 1. The specific peak of PCL carbonyl is seen further to the left in patch 1 because the specific carbonyl peaks of PCL (1722 cm^−1^) and oil (1745 cm^−1^) are at close wavenumbers. The slight left shift (1741 cm^−1^) of the specific peak originating from the PCL polymer in the oil-containing patch (patch 1) also confirms the integration of oil into patch 1. Specific peaks of the components incorporated into the patch, such as polymers (PP-Cl, PEG, PCL) and oil (*H. perforatum*), were observed at similar wavenumbers in the spectra taken for patch 0 and patch 1 after their interaction with the solvent and electrospinning. The absence of significant changes in the wavenumbers of the specific peaks of the components (PP-Cl, PEG, PCL, oil) and the reappearance of the relevant peaks at similar wavenumbers in the FTIR spectra of patch 0 and patch 1 confirm that the chemical structures of the polymers and the oil do not change as a result of their interaction with the solvent.

### 3.2. Morphological Analysis by Scanning Electron Microscope (SEM)

**Figure 3:**
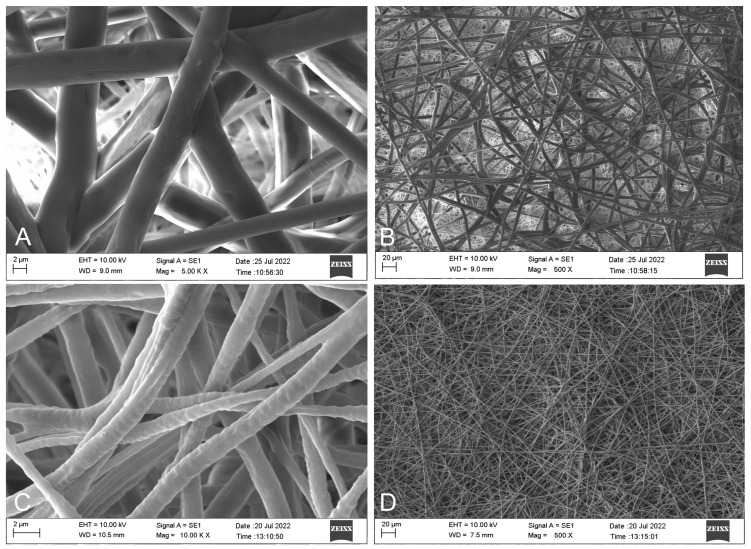
SEM images of patch 0. (**A**) and (**B**) are upper (parietal) layer 5000× and 500× magnifications; (**C**) and (**D**) are lower (visceral) layer 10,000× and 500× magnifications.

**Figure 4:**
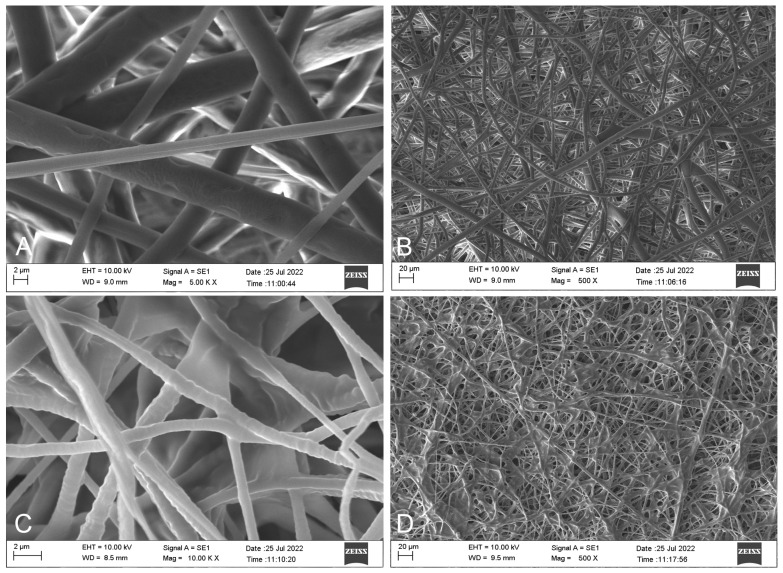
SEM images of patch 1. (**A**) and (**B**) are upper (parietal) layer 5000× and 500× magnifications; (**C**) and (**D**) are lower (visceral) layer 10,000× and 500× magnifications.

SEM images taken from the surfaces were evaluated to examine the surface morphology of the produced hernia patches (Figure 3 and Figure 4). SEM images of the upper (A & B) and lower (B & C) layers of patch 0 without *H. perforatum* oil are presented in Figure 3. Similarly, SEM images of the upper (A & B) and lower (B & C) layers of patch 1 containing *H. perforatum* oil on the visceral side are given in Figure 4.

The SEM images of the parietal side of the herbal oil loaded at the visceral side (patch 1—Figure 4A,B) and parietal side of the oil-free (patch 0—Figure 3A,B) patches consisting of only non-resorbable PP-Cl fibers were similar to expected. They exhibited an extracellular matrix (ECM)-like network structure, which was formed by fibers with irregular thickness and rough surfaces.

The SEM images of the visceral side of the herbal oil loaded at the visceral side (patch 1—Figure 4C,D) and visceral side of the oil-free (patch 0—Figure 3C,D) patches formed by counter electrospun PEG and PCL fibers differed by the inclusion of herbal oil. The PEG fibers in Figure 4C,D exhibited more distinct integration at intersection points.

### 3.3. Density and Porosity

The porosity (porosity%) and density of prosthetic patches are critical parameters for the biocompatibility of these prostheses after implantation. The calculated density and % porosity values for patches 0 and 1 are presented in Table 1. PP-Cl%, PCL%, PEG%, and percent oil indicate the weight percentage amounts (*w*/*w*%) of the components within the patch structure. The V_0_ and V_m_ values indicate the specific volume (cm^3^/g) for the pure polymeric film and the specific volume (cm^3^/g) for the electrospun polymeric patches, respectively. The density and % porosity values for patch 0 were calculated as 0.20 g/cm^3^ and 82%, respectively, while the values for patch 1 were calculated as 0.27 g/cm^3^ and 75%, respectively.

### 3.4. Water Contact Angle

The measurement of the contact angle of the water droplet dropped on the surfaces of materials is essential in terms of determining the water retention (hydrophobic) and water absorption (hydrophilic) properties of the surfaces. In order to examine the hydrophilic/hydrophobic character of the lower (visceral) and upper (parietal) surfaces of patch 0 and patch 1, the images of the water droplets dropped on the patch surfaces are given in Figures S2 and S3, and the average contact angle measurements taken from the surfaces are presented as Table 2.

The average contact angle (θ) values of the upper layers (PP-Cl) of patch 0 and patch 1 were calculated as 96.58° and 108.29°, respectively (Table 2). These values show that the upper layers of patch 0 and patch 1 have hydrophobic character.

The average contact angle measurement values of the lower (visceral) layers of patches 0 and 1, which are made of PEG&PCL fibers and planned to contact the hernia wound area, are 39.34° and 64.04°, respectively. While the visceral surface showed hydrophilic character in both patches, the contact angle value increased as expected in patch 1, which contained herbal oil that caused a slight increase in the contact angle value.

### 3.5. Moisture Retention Capacity (Swelling Tests)

**Figure 5:**
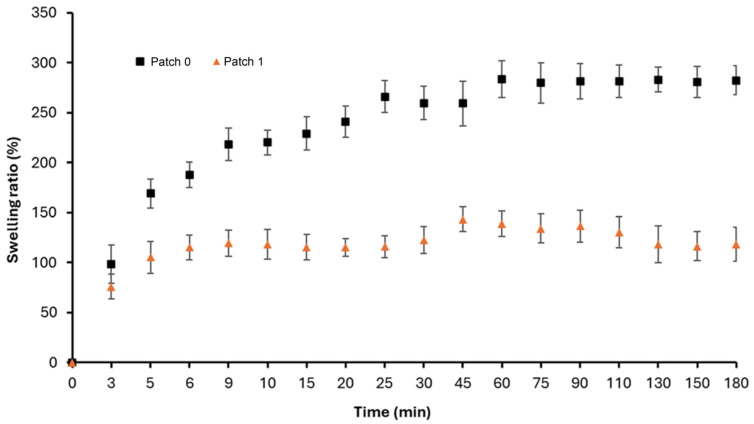
Swelling ratios of patches (0 and 1) over time.

The wettability of the produced patches (patches 0 and 1) was investigated by performing swelling experiments under in vitro conditions. The time-dependent swelling % rates are given in Figure 5.

As can be seen, there is a fast water uptake into the patch by capillary effects until the 60th minute, while the maximum wettability values are reached in both patches at the 180th minute. The maximum swelling values are 282% and 118% for patch 0 and patch 1, respectively. As expected, the moisture retention capacity of patch 0 is higher than that of patch 1.

### 3.6. Time-Dependent Mass Loss

**Figure 6:**
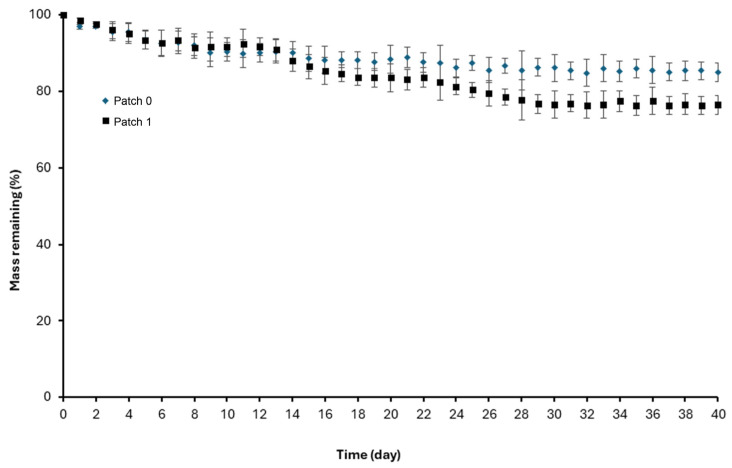
Mass loss of the patches (0 and 1) over time.

In order to measure the time-dependent mass loss of the patches, the initial (W_i_) and final weights (W_s_) of 2 cm × 2cm sections taken from patches 0 and 1 were weighed after being kept in phosphate buffer solution (pH 7.4 and 37 °C) for specific periods, and the dried final weights (W_s_) were calculated with the help of Equation (3). The remaining mass % values are presented graphically in Figure 6. The mass loss experiments for both patches continued for 40 days, and 5 sections were prepared for each day before the experiment. When Figure 6 is analyzed, it is evident that the patches lost mass regularly throughout the 40 days. At the end of the 40th day, the average mass % losses for patches 0 and 1 were calculated as about 15% and about 24%, respectively.

### 3.7. Controlled Release

**Figure 7:**
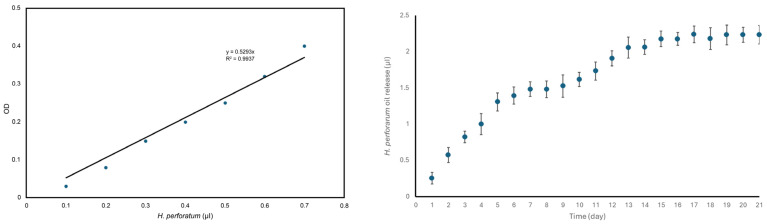
Calibration curve at 225 nm (**left**); release profile from patch 1 for *H. perforatum* (**right**).

Absorbance measurements were taken with a UV–visible spectrophotometer at the wavelength (225 nm) at which the oil gave maximum absorbance from the samples taken from the PBS medium in which the sections taken from patch 1 were kept. The released oil amounts (µL) corresponding to the measured optical density (OD) values were calculated with the help of the trend line equation (y = 0.529x) of the calibration graph and release profile of the oil from patch 1 sketched (Figure 7).

The release profile of the oil was evaluated by analyzing this graph. Within an approximately three-week period where the PEG polymer was almost completely dissolved, it was observed that the oil released by the dissolution of PEG fibers on the patch surface and near-surface regions exhibited nearly a linear release profile in the first week. After the 7th day, the oil release rate decelerated due to the fact that PEG fibers on the patch surface and near-surface regions highly dissolved and released the oil inside. The release rate accelerated again between the 9th and 13th days and became almost constant.

### 3.8. Mechanical Strength

Hernia patches are used to prevent hernia recurrence in the hernia wound area after hernia surgeries and to support the healing process by protecting the wound area. In recent years, with the development of surgical techniques that depend on the location, type, size of the hernia, and the patient’s condition, minimally invasive (laparoscopic) hernia surgeries have been preferred over traditional surgeries. In fact, it has become more critical for the patches, which must be strong and flexible to withstand intra-abdominal pressure and adapt to body movements, to be easily bendable, and to maintain their former shape (elastic) when unpacked in laparoscopic surgeries. Photographs showing that the three-layer, semi-absorbable, polymeric hernia patches produced within this study are bendable are shown in Figure S4.

Tensile tests were performed by taking 5 sections of 0.5 cm × 10 cm each from patches 0 and 1 at room temperature and applying forces varying between 1.5 and 4.5 N at a 50 mm/min constant speed. During the test, force and elongation values of the sections were recorded; stress, strain, elongation at break, and maximum ultimate tensile stress were determined; and yield point and modulus of elasticity (Young’s modulus) values were calculated. The results are presented in Table 3.

The elongation at the break of patch 0, which does not contain oil in the visceral layer, is 222.14 mm, and the maximum tensile stress is 2.1 N/mm^2^. The calculated elongation at break (*σ*:*F*/*A*) and modulus of elasticity (*E*:*σ*/*ε*) values are 1.20 N/mm^2^ and 3.85 ± 1.06 MPa, respectively. The elongation at break of patch 1 containing *H. perforatum* oil in the lower (visceral) layer is 306.99 mm, and the maximum tensile stress is 2.5 N/mm^2^. The calculated elongation at break (*σ*:*F*/*A*) and modulus of elasticity (*E*:*σ*/*ε*) values are 1.04 N/mm^2^ and 2.71 ± 0.07 MPa, respectively.

### 3.9. Cytotoxicity (% Cell Viability, % Apoptosis, % Necrosis)

**Figure 8:**
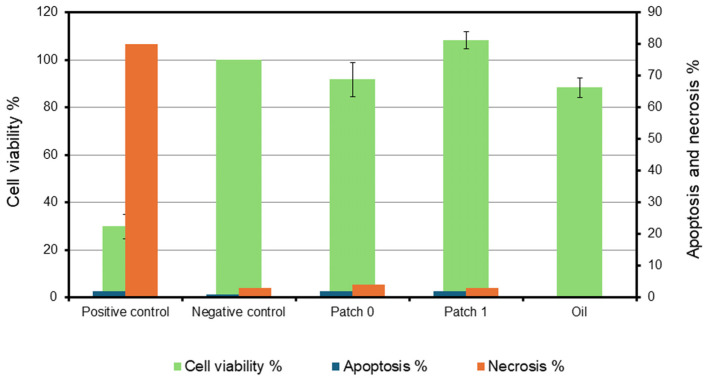
Cytotoxic, apoptotic, and necrotic effects on L929 mouse fibroblast cells.

The MTT test, an intracellular-specific staining test widely used in the literature, was applied to evaluate the cytotoxic effects of the produced hernia patches on L929 mouse fibroblast cells. It was studied for five different groups, with more than four samples in each group. These groups were the group containing only *H. perforatum* oil, the groups consisting of patch sections without oil (patch 0) and containing oil (patch 1), the negative control (NC) group consisting of cells not interacting with any substance or material, and the positive control (PC) group consisting of cells interacting with a solvent known to have toxic effects (DMSO). In addition, all groups except the group containing only *H. perforatum* oil were reconstituted, the double stain test was performed, and the apoptotic and necrotic effects on L929 mouse fibroblast cells were also evaluated. The cell viability %, apoptosis %, and necrosis % values calculated are presented in Table 4 and Figure 8.

When the calculated viability % values, while assuming 100% viability in the negative control group, are evaluated together, the calculated % viability values for patch 0 and patch 1 are 91.79 ± 7.14% and 108.34 ± 3.64%, respectively. It is evident that the patches do not have a cytotoxic effect on L929 mouse fibroblast cells; on the contrary, patch 1, which contains oil, has a slightly proliferative effect on the cells.

### 3.10. Investigation of Cell Adhesion to the Visceral Layer Surface of Patches

**Figure 9:**
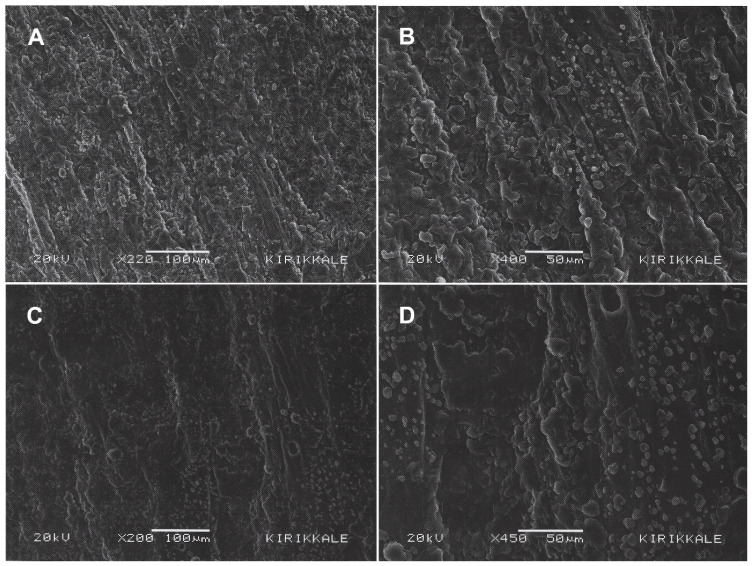
SEM images at different magnifications of patches (patches 0 and 1) cultivated with L929 mouse fibroblast cells: (**A**) patch 0 220×; (**B**) patch 0 400×; (**C**) patch 1 200×; (**D**) patch 1 450×.

L929 fibroblast cells were seeded on the lower (visceral) sides of the patches in a suitable medium, and the visceral patch surface and cells were cultivated under cell culture conditions for 2 days. Then, the presence of any cell adhesion and proliferation on the surfaces was evaluated by investigating the SEM images taken from the visceral surfaces that were left to dry after incubation (Figure 9).

When the SEM images in Figure 9 are evaluated, it is observed that, unlike Figure 3 and Figure 4, the fibrous structure is disrupted due to the dissolution of the PEG fibers in contact with the liquid medium, and there is no cell-like structure attached or proliferated on the surfaces due to the dissolution of the PEG fibers. This situation shows that the potential of the patches to adhere to the internal organs in the hernia region is not high for in vivo applications.

## 4. Discussion

Within the scope of this study, chemical structure analysis, surface morphology, density and porosity values, swelling capacity (wettability), water contact angle measurements (surface hydrophilicity/hydrophobicity), time-dependent mass loss, mechanical strength, the release profile of *H. perforatum* oil from patch 1, and the in vitro cytotoxic/apoptotic/necrotic effects on L929 mouse fibroblast cells of the three-layered semi-absorbable composite hernia patches (patches 0 and 1) produced were evaluated by various characterizations to emphasize the potential of tri-layered semi-resorbable patches to be used in hernia therapy. Chemical structure analyses confirmed that the *H. perforatum* oil was successfully loaded into patch 1 and that the polymers’ chemical structure was not affected due to their interactions with the solvent or electrospinning environment.

SEM analyses confirmed the formation of fibers with irregular thickness and rough surfaces that exhibited an ECM-like structure. Thus, it may induce tissue compatibility with the surrounding tissues in vivo from a biomimetic perspective. During the patch production phase, to minimize the disadvantage of polypropylene, which is difficult to dissolve and electrospun at room temperature, an electrospun polypropylene membrane could be obtained by using a chlorinated-form PP-Cl, which can be more easily dissolved at room temperature, form a more conductive solution, and thus enable electrospinning. However, the optimization of the electrospinning conditions of this polymer was not easy. Although the flow rate, solution concentration, applied potential difference, and the distance between the needle tip and the drum collector were optimized, PP-Cl fibers with different thicknesses may be formed during electrospinning due to external factors that cannot be fully controlled, such as the humidity of the spinning atmosphere and the conductivity of the air. As confirmed by the SEM images, this resulted in the achievement of a construct more similar to the ECM, and this may increase the patch’s compatibility with the surrounding tissue under in vivo conditions on the parietal side. However, instead of a mechanically strong but though layer consisting of uniform thick fibers, a layer formed of thick and thin fibers together can be mechanically strong and more flexible. Therefore, although attempts were made to create fibers of equal thickness, it is thought that the obtained structure may be more advantageous in hernia patch applications due to the preference for lighter, durable, and flexible patches. When the SEM images of patches 0 and 1 are compared, the inclusion of oil in PEG resulted in rougher and better-integrated fibers at the intersection points. This may be due to the fact that the hydrophobic oil is localized more heterogeneously instead of being distributed homogeneously within the hydrophilic PEG polymer. Due to the flow rates on the visceral surfaces, it is thought that the thick fibers are PCL and the thinner fibers are PEG fibers. In general, the fact that the fibrous structure consisting of intertwined fibers in all images of the lower- and upper-layer surfaces was formed without any pilling, etc., problems originating from the electrospinning conditions show that the electrospinning technique was applied successfully.

The results of the density and porosity evaluations showed that the addition of *H. perforatum* oil slightly increased the patch density and slightly decreased the percent porosity of the patch. Similarly, the SEM images (C and D) of patch 1 in Figure 4 confirm that the oil trapped in the fiber reduces the pore size and increases the density. The patches’ low density and high porosity, an advantage of the electrospinning technique, reduce the risk of immune reactions in the surrounding tissue that may occur in the short and long term in clinical applications. The values for both patches were similar to the study by Miao et al. [25].

If the angle (θ) made by the dropped water droplet on the patch surface is less than 90° (θ < 90°), it is stated that the surface is wetted and the material is hydrophilic; if it is greater than 90° (θ > 90°), it is noted that the surface is not wetted, and the material is hydrophobic [26]. However, if the droplet makes angles narrower than 10° (θ < 10°) with the surface, the material surface can be said to be super-hydrophilic, or if it makes angles wider than 150° (θ > 150°), the material surface can be said to be super-hydrophobic. Evaluations of the contact angles revealed that the top layers of patches 0 and 1 exhibited a hydrophobic character. The hydrophilic nature of biomaterials increases the adhesion of cells in the surrounding tissue to the material’s surface, while the opposite can be observed on excessively hydrophilic surfaces. On the other hand, a hydrophobic nature makes adhesion difficult, while excessive hydrophobicity can increase the possibility of generating an immune response. Therefore, in biomaterial design, the surface properties of the parts that will remain not biodegraded are critical in minimizing some problems that may occur in long-term use. If cells are desired to penetrate and adhere to the surface and the material of biodegradable materials, such as tissue scaffolds, etc., and to form a new tissue by proliferation, a balanced hydrophilic character is desired for these materials. On the contrary, while functional compatibility and biocompatibility with the surrounding tissue are desired for implants, such as hernia patches, stents, heart valves, etc., a negative interaction such as inhibiting the functional properties of the material by being wrapped by the connective tissue or sticking to tissues and organs is not desired. Therefore, it is advantageous for the nondegradable upper parts of the patches produced within the scope of this study to exhibit a balanced hydrophobic character. The hydrophobic oil reduced the surface hydrophilicity of patch 1. The hydrophilic PEG fibers accompanying the more hydrophobic PCL fibers ensured the surface was hydrophilic. It is thought that the PEG fibers, which are planned to dissolve in a short time and release *H. perforatum* oil in a controlled manner during the acute process while the hernia wound is healing, will not cause cell adhesion on the surfaces due to dissolution, although they show hydrophilic properties. Although L929 mouse fibroblast cells interacted with the surface under cell culture conditions for 2 days under in vitro conditions, the SEM images taken from the dried surfaces after the experiment support this situation, as the fibrous structure deteriorated due to dissolution in the liquid medium and the absence of cell-like structures on the surfaces. The fact that PCL fibers, which degrade in a much longer time compared to the PEG polymer, have a more hydrophobic character due to the structure of the PCL polymer suggests that there will be no risk of long-term adhesion to tissues and organs in the lower layer as in the upper layer (PP-Cl).

Generally, it is essential to provide a controlled moisture balance in the wound area so that wounds can heal quickly without unwanted conditions such as infection [27]. It is advantageous for biomaterials in the wound area to absorb excess wound exudate. Although wound exudate formation in open wounds increases the risk of infection, it is not desirable to absorb wound exudate in the acute process completely. Because bioactive agents that support wound healing and new tissue formation are delivered to the wound area via body fluids, swelling in the wound area is present in cases such as invasive procedures like surgery, impact, and injury. Providing a controlled moisture balance in the wound area is related to the water retention capacity of the patch.

The herbal oil integrated into the patch structure on the visceral side has both a hydrophobic character and reduced patch porosity, as seen in the SEM images (Figure 4) and porosity (Table 1), significantly reducing the moisture retention capacity in patch 1 compared to patch 0. However, as an advantage of the electrospinning technique in both patches, the moisture retention capacity is high due to increased capillary effects due to high porosity. As a result, it can be said that both patches (patches 0 and 1) have sufficient moisture retention capacity for wound healing when compared to the relevant literature [28,29]

The mass losses of patches 0 and 1 were about 15% and 24%, respectively, and it is thought that the mass losses are due to the dissolution of PEG fibers in the absorbable lower (visceral) layers of the patches in a short period of approximately three weeks. Especially after the 21st day, the mass loss rate almost stopped due to the large-scale dissolution of the PEG polymer. The fact that the remaining mass % values calculated in patch 1 were lower compared to patch 0 during the mass loss experiments is due to the entrapment of *H. perforatum* oil in the PEG fibers that dissolved in patch 1. As herbal oil was also a component in the total mass of the patch, it increased the total mass loss from the patch while it was released together with the dissolution of the PEG fibers. The release profile of the oil entrapped in PEG fibers exhibited a controlled manner (Figure 7), and the release almost wholly stopped on the 21st day. PCL fibers, which are also among the parts of the patch that degrade over time, degrade in a much more extended period (2–3 years [30]) compared to PEG polymers, depending on their molecular weight, and as expected, the mass losses resulting from the degradation of PCL fibers were not reflected in the 40-day mass loss experimental data. Thus, PCL fibers provide mechanical support to PP-Cl fibers in the middle and lower layers until the healing process is entirely completed; then, they will completely degrade and significantly reduce the total patch weight. Accordingly, the risk of long-term foreign body reactions is diminished, while the non-absorbable upper (parietal) PP-Cl layer, which prevents hernia recurrence, can continue to protect the area where hernia surgery has been performed permanently.

The reason why the oil is trapped inside the PEG fibers during the production process, instead of being incorporated into the fibers of the patches after production by methods such as dripping from the surface or spraying, is to prevent excessive oil accumulation in the hernia wound area in the first few days and the occurrence of undesired circumstances related to this and to ensure the controlled release of the oil with the dissolution of the PEG fibers in order to support wound healing.

The release profile observed (Figure 7) can be attributed to the release of tiny oil droplets from the fibers dissolved in the inner parts of the lower layer of the patch, which come together and cluster in the layer, and the egress from the pores of the patch due to the weakened capillary and diffusion barrier effects together with the significant deterioration of the surface. At the end of the two weeks, the oil release rate decelerated considerably due to the decrease in undissolved PEG fibers in the patch structure, and the amount of oil in the medium almost did not change. In general, it can be said that the oil was released with a controlled profile by spreading over time rather than being released suddenly throughout the process.

Knitted patches (e.g., Marlex^®^, Dacron^®^, and Prolene^®^) provide more flexibility and larger pores than woven patches but are weaker in strength. For example, the mechanical strengths of Marlex^®^, Prolene^®^, and Surgipro^®^, among the most commercially preferred patches, are 2.59 MPa, 2.46 MPa, and 3.25 MPa, respectively [11]. Standard tensile tests were performed on the patches produced in this study, and the mesh strengths were determined as 3.85 ± 1.06 MPa for patch 0 and 2.72 ± 0.07 MPa for patch 1. These values are comparable to commercially available hernia meshes. The produced patches are also capable of easily withstanding physiological stresses in living organisms, just like commercial patches. Meshes used to repair abdominal hernias must have at least 180 mmHg (20 kPa) strength to withstand physiological stresses [4,5,6]. Even the lightest commercial patches can withstand up to twice this stress without bursting (e.g., Vypro^®^ burst pressure 360 mmHg). The standard heavyweight polypropylene patch is reported to have a bursting strength of 6 to 10 times this calculated stress value [6]. Since the integration of herbal oil into the visceral layer slightly affected the porosity, density, and fibrous morphology, it slightly reduced the elasticity by decreasing the modulus of elasticity while increasing the ultimate tensile stress (UTS) and elongation at break.

In general, it can be said that the three-layered semi-absorbable composite patches are flexible, durable, and can provide the ease of operation that surgeons need [4,11].

The cell viability % values of the patches 0 and 1 were found to be 91.79 ± 7.14% and 108.34 ± 3.64%, respectively, and it is evident that the patches do not have a cytotoxic effect on L929 mouse fibroblast cells. It can be said that patch 1, which contains oil, has a slightly proliferative effect on the cells. This situation is thought to support the wound-healing process in the hernia region in clinical applications. In addition, it is evident that *H. perforatum* oil does not have a cytotoxic effect on the cells. However, the data obtained from the double staining test show that the patches do not have a significant apoptotic or necrotic effect on L929 mouse fibroblast cells when compared to the negative control group, meaning a positive aspect to be biocompatible. The SEM images obtained after 2 days of cultivation of L929 fibroblast cells on the visceral side revealed that the fibrous structure was disrupted due to the dissolution of PEG fibers on the surfaces. Thus, cell adhesion to the surface was prevented.

## 5. Conclusions

Semi-absorbable composite hernia patches consisting of three layers designed for treating abdominal hernias have been successfully produced, and the findings obtained from in vitro characterization studies have been evaluated. It has been concluded that both patches have the potential to be used in clinical applications as a new-generation semi-absorbable hernia patch with appropriate densities, sufficient flexibility, non-cytotoxicity, reduced risk of tissue adhesion, and wound healing-supporting properties. Therefore, this study is a detailed preliminary study for further research, such as in vivo and clinical studies. A therapeutic herbal oil has been integrated into hernia patches for the first time with this study; therefore, it may open a new window to hernia mesh research.

## Figures and Tables

**Table 1:** Specific volume, density, and % porosity values of patch 0 and patch 1.

Patch	PEG (*w*/*w*%)	PCL (*w*/*w*%)	PP-Cl (*w*/*w*%)	Oil (*w*/*w*%)	Specific Volume (V_0_) (cm^3^/g)	Specific Volume (V_m_) (cm^3^/g)	Density (g/cm^3^)	Porosity (%)
0	0.137	0.517	0.345	0	0.894	5.102 ± 1.071	0.20 ± 0.04	82 ± 4
1	0.104	0.392	0.261	0.244	0.932	3.703 ± 0.967	0.27 ± 0.06	75 ± 6

**Table 2:** Average contact angle values of the bottom and top layer surfaces of patches 0 and 1.

Patch	Bottom (Visceral) Layer (θ)	Top (Parietal) Layer (θ)
0	39.34° ± 3.93°	96.58° ± 1.82°
1	64.04° ± 5.84°	108.29° ± 5.87°

**Table 3:** Mechanical test results of patches (0 and 1).

Patch	Elongation at Break (mm)	Ultimate Tensile Stress (N/mm^2^)	Yield Point (N/mm^2^)	Young Modulus (MPa)
0	222.14	2.1	1.20	3.85 ± 1.06
1	306.99	2.5	1.04	2.71 ± 0.07

**Table 4:** Cytotoxic, apoptotic, and necrotic effects on L929 mouse fibroblast cells.

Groups	Apoptosis%	Necrosis%	Cell Viability%
Positive control (DMSO)	2	80	29.83 ± 5.06
Negative control (cells only)	1	3	100 (Reference)
Patch 0	2	2	91.79 ± 7.14
Patch 1	2	3	108.34 ± 3.64
*H. perforatum* oil	-	-	88.36 ± 4.15

## Data Availability

Data are contained within the article and supplementary materials.

## Supplementary Materials

A supplementary file containing four figures is attached. The following supporting information can be downloaded at: https://www.mdpi.com/article/10.3390/polym17020240/s1, Figure S1: Composite patches produced by electrospinning on a rotating drum collector; Figure S2: Representative contact angle measurement images of Patch 0 (A) bottom and (B) top layer surfaces; Figure S3: Representative contact angle measurement images of Patch 1 (A) bottom and (B) top layer surfaces; Figure S4: Flexible polymeric patch strips.
