# Supplementary material for: Production and Characterization of H. perforatum Oil-Loaded, Semi-Resorbable, Tri-Layered Hernia Mesh
Source: Polymers (Basel). 2025 Jan 19;17(2):240. doi: 10.3390/polym17020240 (PMC11768532; doi:10.3390/polym17020240)
Supplement: Supplementary file 1 [file polymers-17-00240-s001.zip › polymers-3406004-supplementary.pdf]

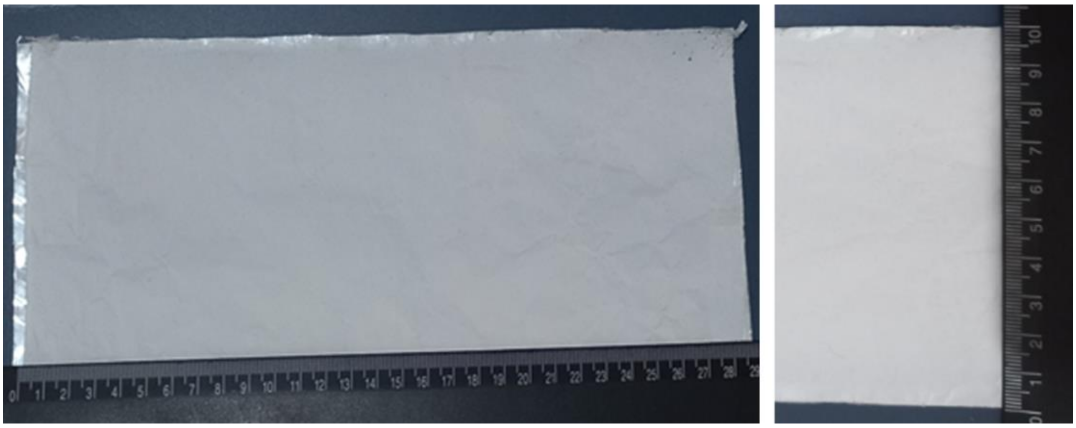

Figure S1. Composite patches produced by electrospinning on a rotating drum collector

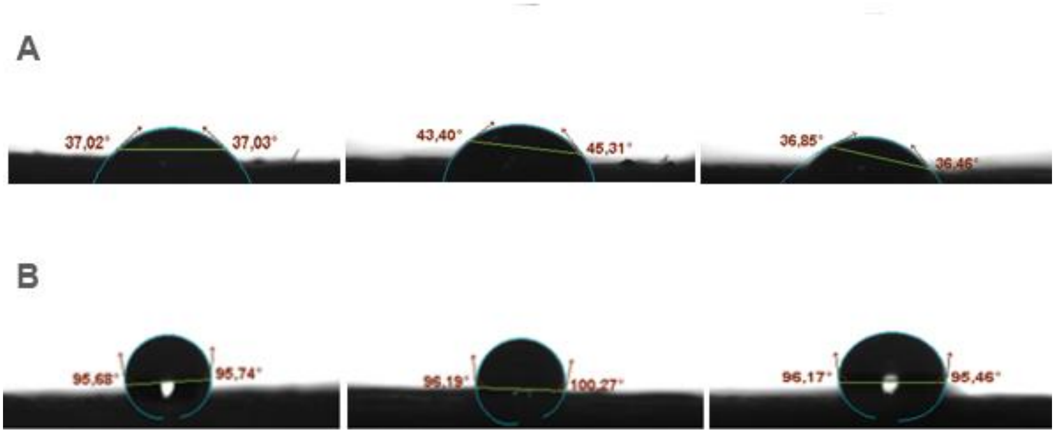

Figure S2 Representative contact angle measurement images of Patch 0 (A) bottom and (B) top layer surfaces,

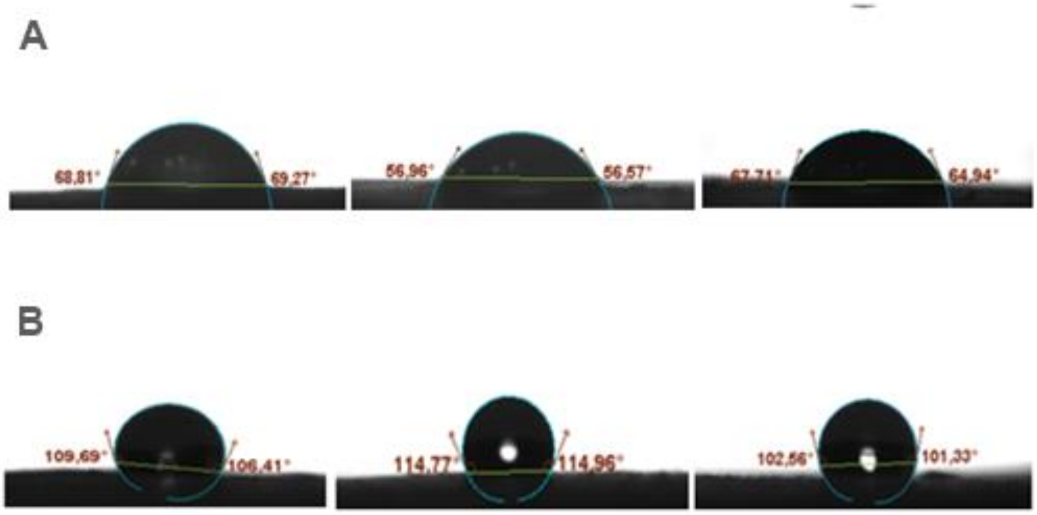

Figure S3 Representative contact angle measurement images of Patch 1 (A) bottom and (B) top layer surfaces

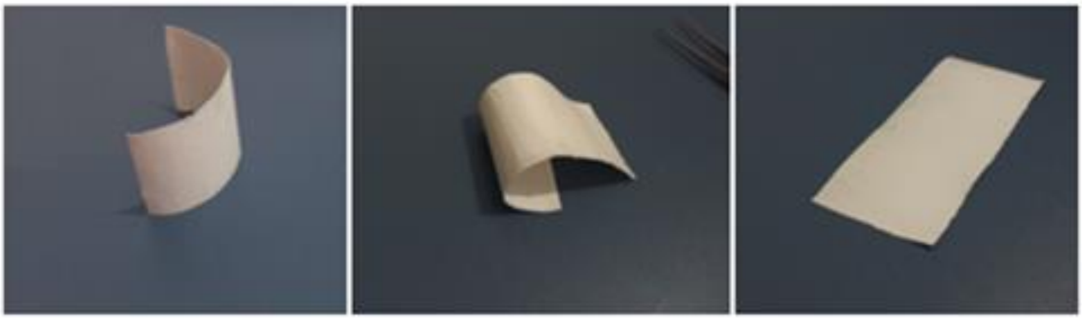

Figure S4. Flexible polymeric patch strips
